# Cancer Vaccines Based on Fluorine-Modified KH-1 Elicit Robust Immune Response

**DOI:** 10.3390/molecules28041934

**Published:** 2023-02-17

**Authors:** Yang Liu, Bohan Li, Xiujing Zheng, Decai Xiong, Xinshan Ye

**Affiliations:** 1 State Key Laboratory of Natural and Biomimetic Drugs, School of Pharmaceutical Sciences, Peking University, Xue Yuan Rd. No. 38, Beijing 100191, China; 2 The NMPA Key Laboratory for Quality Research and Evaluation of Carbohydrate-Based Medicine, Shandong University, 27 Shanda Nanlu, Jinan 250100, China

**Keywords:** tumor-associated carbohydrate antigens, KH-1, fluoro-modification, tumor vaccine

## Abstract

KH-1 is a tumor-associated carbohydrate antigen (TACA), which serves as a valuable target of antitumor vaccines for cancer immunotherapies. However, most TACAs are thymus-independent antigens (TD-Ag), and they tend to induce immunological tolerance, leading to their low immunogenicity. To overcome these problems, some fluorinated derivatives of the KH-1 antigen were designed, synthesized, and conjugated to the carrier protein CRM197 to form glycoconjugates, which were used for immunological studies with Freund’s adjuvant. The results showed that fluorine-modified *N*-acyl KH-1 conjugates can induce higher titers of antibodies, especially IgG, which can recognize KH-1-positive cancer cells and can eliminate cancer cells through complement-dependent cytotoxicity (CDC). The trifluoro-modified KH-1-TF-CRM197 showed great potential as an anticancer vaccine candidate.

## 1. Introduction

Some aberrant glycans are overexpressed on the surface of tumor cells due to the abnormal expression of glycosidases and glycosyltransferases, and they are correlated with tumor cell adhesion and metastasis. These aberrant glycans are called tumor-associated carbohydrate antigens (TACAs). They are important immunotherapeutic targets against cancers [1,2,3]. Unfortunately, most TACAs are thymus-independent antigens, and they are also expressed by normal cells, leading to their low immunogenicity [4,5]. To enhance the immunogenicity of TACAs, they are usually conjugated with immunogenic carriers, and unnatural TACA analogues are also introduced through the modification of TACAs [6,7,8,9,10,11].

The KH-1 antigen is one of the TACAs found in human colon cancer cells. It is a branched nonasaccharide glycolipid containing two TACA epitopes (the tetrasaccharide of the Le^y^ antigen and the trisaccharide of the Le^x^ antigen) [12,13,14]. It is barely expressed by normal tissues but highly expressed by adenocarcinoma cells [15]. Compared with the low incidence (5–10%) of oncogenes and the deletion (<50%) of tumor-suppressing gene products, the incidence of the extended Le^y^ antigens expressed in colorectal cancer is up to about 90% [5]. Therefore, the KH-1 antigen is a potential immunotherapeutic target against adenocarcinoma. Tumor vaccines based on the KH-1 antigen have been proven to induce IgM and IgG antibodies against KH-1 and to show great safety due to low cross-reactivity with the Le^x^ antigen, which is also expressed by neutrophilic granulocytes [16,17]. However, as a self-antigen, the natural KH-1 antigen regularly leads to immunological tolerance, which is a major challenge in carbohydrate-based vaccine design.

The chemical synthesis of KH-1 is extremely valuable and challenging because it cannot be obtained in large quantities from natural resources and has a complex structure. At present, several research groups have completed the total synthesis of KH-1 through different strategies, in which Guo and our group have achieved relatively high synthetic yields due to the use of a one-pot glycosylation protocol [18,19,20,21,22]. Reliable and efficient synthesis provides support for the study of biological activities.

**Figure 1:**
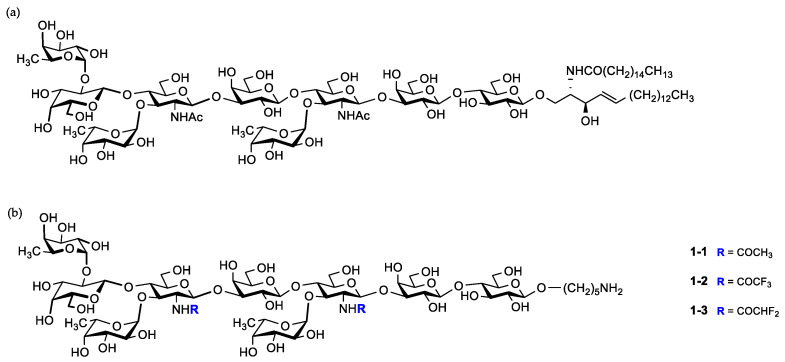
(**a**) Native KH-1 antigen; (**b**) fluorine-modified and unmodified KH-1 derivatives in this work.

As fluorine has a similar atomic radius and lipophilicity to hydrogen and is a trace element for humans, fluoro-modifications of TACAs have been proven to increase the antitumor immunogenicity of TACAs. Previously, our group conducted a systematic study of fluorine-modified TACAs, such as STn and Globo H [23,24,25,26]. Compared with unmodified glycoconjugates, fluorine-modified glycoconjugates induced higher titers of IgG antibodies. Fluoro-modifications of TACAs can enhance the CDC effect of antisera on tumor cells. Based on our previous study, we hope to improve the immunogenicity of KH-1 through fluorinated modifications. Herein, we want to report the design and synthesis of these fluorine-modified KH-1 derivatives (Figure 1) and evaluate the immunogenicity of their corresponding glycoconjugates.

## 2. Results and Discussion

### 2.1. Synthesis of KH-1 Antigen and Its Fluorinated Derivatives

**Scheme 1:**
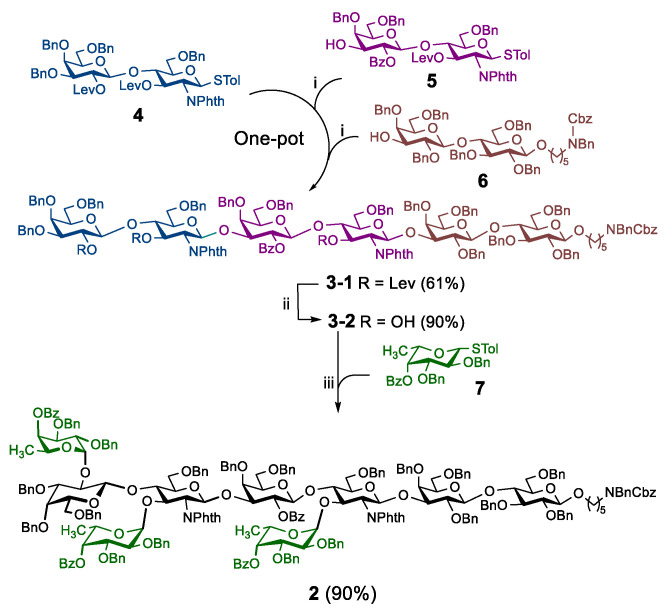
Photo-induced synthesis of the fully protected KH-1 core nonasaccharide: (**i**) UV, 4 Å molecular sieves, Umemoto’s reagent, Cu(OTf)_2_, TTBP, −72 °C, 20 min; then acceptor, −72 °C to rt, 1.5 h; (**ii**) H_2_NNH_2_·HOAc, CH_3_OH/THF (1:10); (**iii**) UV, 4 Å molecular sieves, Umemoto’s reagent, Cu(OTf)_2_, TTBP, −50 °C, 4 min; then acceptor, −50 °C to rt, 2.5 h.

As described in our previous report [27], three disaccharide building blocks **4**, **5**, and **6** and one fucose building block **7** were prepared through dozens of steps. Next, the combination of photo-induced strategy [28,29] with a preactivation-based one-pot glycosylation protocol [30] was employed to obtain a linear hexasaccharide **3-1**, which was subjected to partial deprotection to yield hexasaccharide **3-2**. After screening the reaction conditions, the photo-induced fucosylation was finally realized, providing the KH-1 core nonasaccharide **2** in high yield (Scheme 1) [27].

**Scheme 2:**
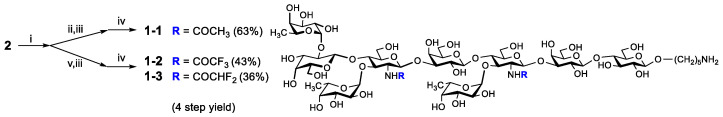
Deprotection and synthesis of fluorinated derivatives: (**i**) H_2_NCH_2_CH_2_NH_2_/n-BuOH (1:3), 120 °C; (**ii**) Ac_2_O/MeOH; (**iii**) LiOH, MeOH/THF (4:1), 80 °C; (**iv**) Pd(OH)_2_, H_2_, CH_2_Cl_2_/MeOH/H_2_O (3:3:1); (**v**) DMAP, ROCH_3_/MeOH.

The global deprotection of **2** in four successive steps was carried out to afford compound **1-1** smoothly. In order to generate the fluorinated derivatives, a series of conditions were attempted for the fluoroacetylation step. For the trifluoroacetylation reaction, the reaction conditions were assessed (Table S1), and the optimized conditions were as follows: methyl trifluoroacetate as the fluorinated reagent, 4-dimethylaminopyridine (DMAP) as the base, dry methanol as the solvent, and under room temperature for 48 h. Using similar fluoroacetylation conditions and deprotection steps, the fluorinated compounds **1-2** and **1-3** were prepared (Scheme 2). We also sought to obtain the monofluorinated derivative, but unfortunately, it was not successful due to the restrictions in reagent purchase and preparation.

### 2.2. Synthesis of Glycoconjugates

**Scheme 3:**
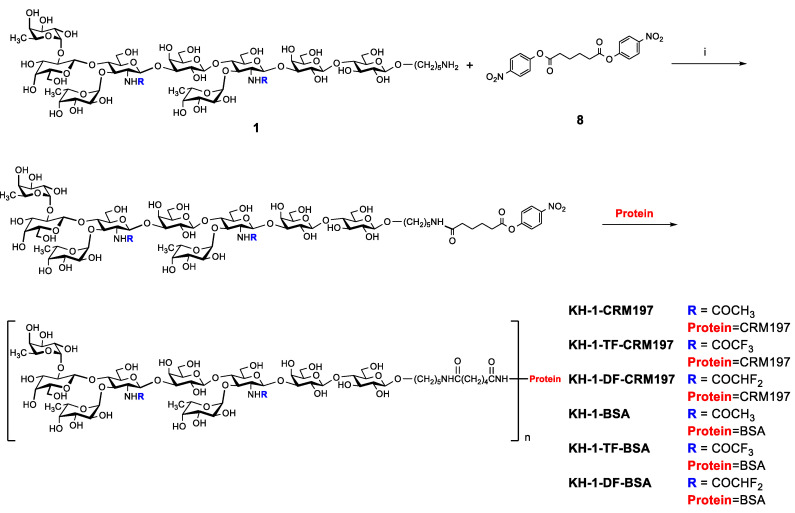
Synthesis of glycoconjugates: (**i**) DMF, (C_2_H_5_)_3_N.

Diphtheria toxoid cross-reactive material 197 (CRM197) is a vaccine carrier approved by the FDA [31]. Globo H-CRM197 has been proven to induce higher titers of the IgG antibody than Globo H-KLH [32]. Moreover, it is effortless for keyhole limpet hemocyanin (KLH) to form polymers, which may disturb the quality control of vaccines [33]. Therefore, CRM197 was chosen as the carrier of the KH-1 antigen. The three nonasaccharide molecules mentioned above were covalently linked to the CRM197 protein and the BSA protein via a known linker [34], respectively (Scheme 3). Finally, six glycoconjugates were obtained. The carbohydrate loading levels were determined using the mass spectra analysis (Table 1 and Figures S1–S8).

### 2.3. Evaluation of the Antibodies

With the glycoconjugates in hand, the immunological properties of these conjugates were evaluated in mice. Groups of six female BALB/c mice were vaccinated four times at biweekly intervals with the CRM197 conjugates using Freund’s adjuvant. After immunization, antisera were obtained and used for KH-1 antibody detection. Anti-KH-1 or anti-modified-KH-1 titers were firstly evaluated using ELISA (enzyme-linked immunosorbent assay) plates coated with KH-1-BSA or modified KH-1-BSA, using the pooled antisera of all immunized mice after the third or fourth vaccination (Table 2). We found that KH-1-DF-CRM197 and KH-1-TF-CRM197 induced a stronger KH-1-specific antibody response and elicited higher titers of anti-KH-1 IgG and IgM antibodies than KH-1-CRM197 after the third and fourth vaccination. The sera from preimmunized mice showed no titer of the IgG or IgM antibodies against the KH-1 antigen. The IgG/IgM ratio of KH-1-TF-CRM197 was particularly increased. For tumor immunotherapy, the IgG response is more functional than IgM on account of their properties, such as affinity maturation and immunological memory [35,36]. In addition, KH-1-DF-CRM197 and KH-1-TF-CRM197 elicited higher titers of anti-modified-KH-1 IgG antibodies than KH-1-CRM197, which again proved that fluoro-modifications of KH-1 can increase its antibody response.

**Figure 2:**
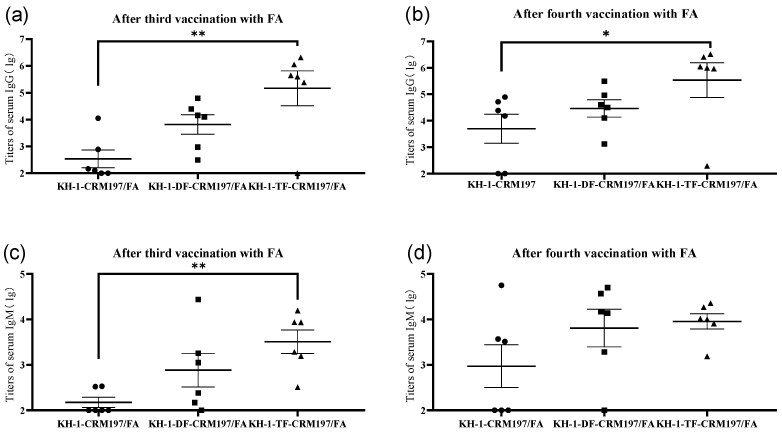
Serum antibody titers against KH-1-BSA in an individual mouse vaccinated with KH-1-CRM197, KH-1-DF-CRM197, or KH-1-TF-CRM197 after the 3rd (**a**,**c**) and 4th (**b**,**d**) vaccinations. Each data point represents the average titer of two detections in an individual mouse, and the black line in each series represents the mean titer of six mice in each group. The data of IgG titers were plotted in the form of log 10. * *p* < 0.05, ** *p* < 0.01.

The anti-KH-1 titers of IgG and IgM for an individual mouse after the third and fourth vaccination were also detected using ELISA (Figure 2). The IgG and IgM titers of modified KH-1 conjugates, especially KH-1-TF-CRM197, were higher than those of KH-1-CRM197. This demonstrated that the modified KH-1 conjugates, especially KH-1-TF-CRM197, produced higher titers of anti-modified-KH-1 antibodies with cross-reactive efficiency for KH-1, resulting in an increase in the anti-KH-1 IgG and IgM antibodies.

**Figure 3:**
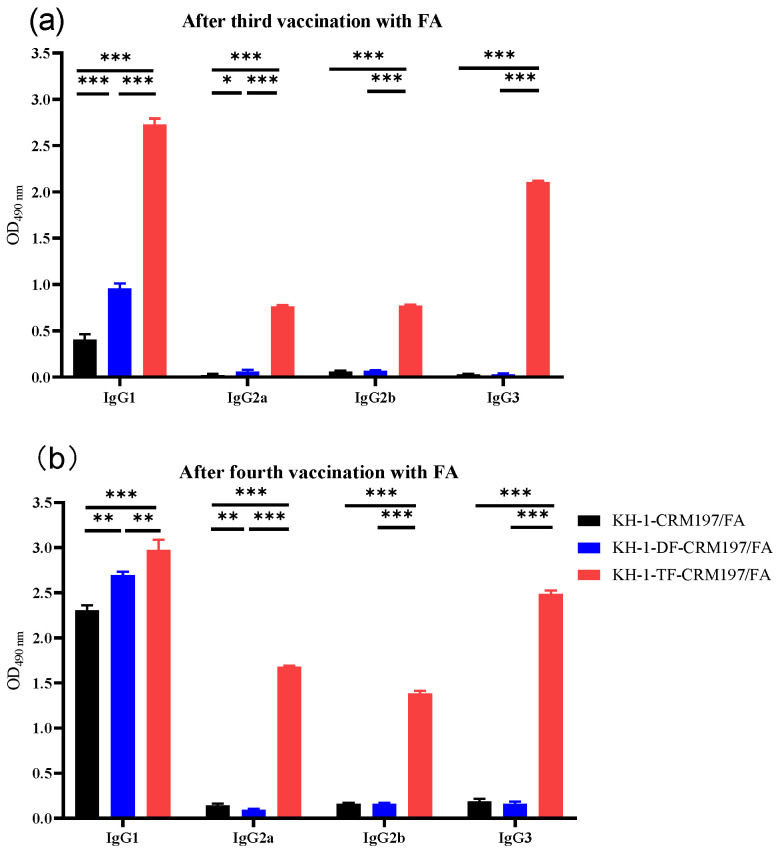
IgG subtypes after the third (**a**) and fourth (**b**) vaccinations with KH-1-KLH, KH-1-DF-KLH, and KH-1-TF-KLH with Freund’s adjuvant using ELISA with a 1:1000 dilution of pooled sera. Results represent the mean ± SEM of three independent experiments. One-way ANOVA analysis was performed. * *p* < 0.05, ** *p* < 0.01, *** *p* < 0.01.

The IgG subtypes in antisera after the third and fourth vaccinations were also detected with ELISA (Figure 3). Immunized with Freund’s adjuvant, KH-1-TF-CRM197 elicited significantly more anti-KH-1 IgG1, IgG2a, IgG2b, and IgG3 antibodies than the unmodified KH-1-KLH. These results indicated that a mixed Th1/Th2 response was elicited by KH-1-TF-CRM197 because IgG1 antibodies are usually generated via the Th2 response, while activated Th1 cells may generate a prominent IgG2a response [37].

### 2.4. Recognition of KH-1-Positive Tumor Cells with IgG Antibodies in Antisera

**Figure 4:**
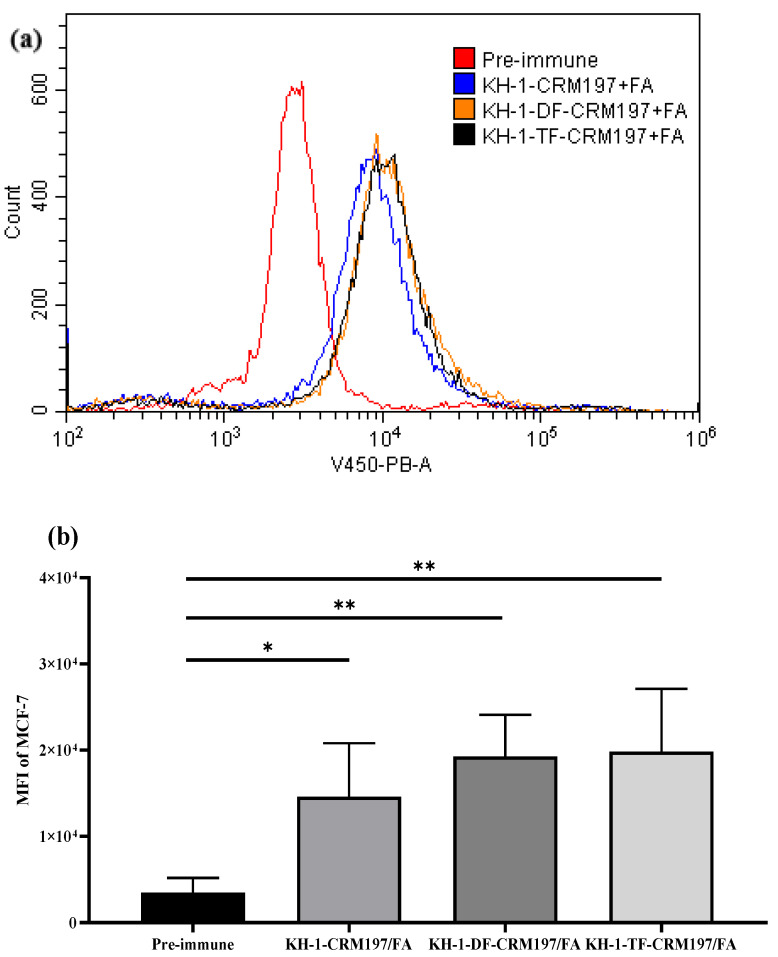
Recognition of human tumor cells by IgG antibodies in antisera: (**a**) serological IgG analysis results on MCF-7 human breast cancer cells after the fourth immunization via flow cytometry; (**b**) mean fluorescence intensity (MFI) of the IgG antibody’s binding to MCF-7 cells. Results are representative of three independent experiments. One-way ANOVA analysis was performed. * *p* < 0.05, ** *p* < 0.01.

Flow cytometry was used to evaluate the capabilities of antisera induced by KH-1-CRM197, KH-1-DF-CRM197, or KH-1-TF-CRM197 to recognize the native KH-1 antigens of cancer cells (Figure 4). KH-1-positive MCF-7 human breast cancer cells were used as target cells, and the preimmune serum was used as a negative control [38]. The results showed that the IgG antibodies elicited by the three glycoconjugates could bind well with MCF-7 cells on which KH-1 antigens were overexpressed. This indicated a great cross-reactivity of antisera elicited by the fluorine-modified KH-1-CRM197 to the native KH-1 antigen on tumor cells, which is the foundation of the antitumor activities of fluorine-modified KH-1-CRM197. Furthermore, the IgG antibody induced by fluorine-modified glycoconjugates (KH-1-DF-CRM197 and KH-1-TF-CRM197) showed a stronger binding affinity to MCF-7 cells than the unmodified KH-1-CRM197, which was consistent with the trend of the IgG titer results.

### 2.5. Complement-Dependent Cytotoxicity of Antisera Induced by Vaccines

**Figure 5:**
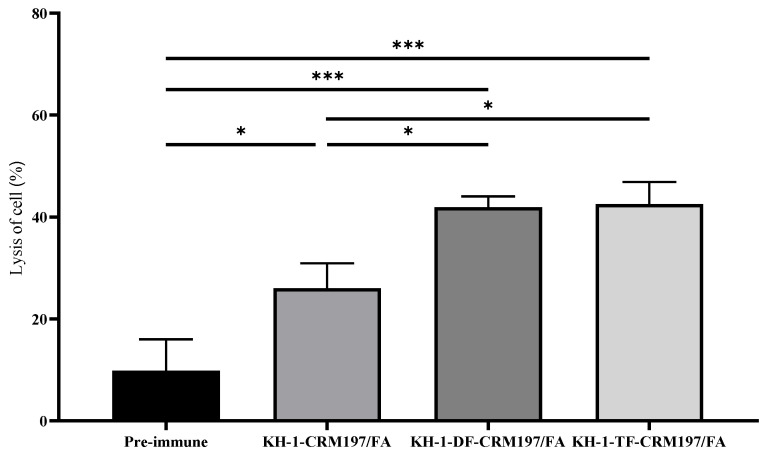
Antibodies elicited by glycoconjugates mediate complement-dependent cytotoxicity (CDC) to eliminate KH-1-positive MCF-7 cancer cells. Cell lysis of pooled sera (1:20 dilution) after the 4th vaccination. Data are the mean ± SEM of three independent experiments. Cytotoxicity is shown as percentage cell lysis determined by the lactate dehydrogenase (LDH) assay. Results are representative of three independent experiments. One-way ANOVA analysis was performed. * *p* < 0.05, *** *p* < 0.01.

According to previous reports [24,39,40], complement-dependent cytotoxicity (CDC) is a potent mechanism of cell killing. Therefore, CDC was studied to determine whether the immune response provoked by the fluorine-modified KH-1-CRM197 is useful for cancer immunotherapy. We found that the antisera elicited by the three glycoconjugates after the fourth vaccination were able to increase the lysis of KH-1-positive MCF-7 cells compared with that in the preimmune group (Figure 5). Fluoro-modifications on KH-1 can provoke the CDC of antisera against tumor cells. This might be associated with the fact that the fluorine-modified KH-1-CRM197 produced more IgG3 and IgM antibodies against the KH-1 antigen, which mainly acts via the complement [41].

Taken together, these results reveal that fluoro-modifications of the KH-1 antigen can improve its immunogenicity. This might be attributed to the increased enzymatic stability, enhanced affinity to T-cell receptor (TCR), and exogenous properties of fluoride antigens [11].

## 3. Materials and Methods

### 3.1. Chemical Synthesis

The reactions were carried out in oven-dried glassware. All reactions were carried out under anhydrous conditions with freshly distilled solvents under a positive pressure of argon unless otherwise noted. Umemoto’s reagent refers to *S*-(trifluoromethyl)dibenzothiophenium tetrafluoroborate. The reactions were monitored using thin-layer chromatography (TLC) on silica-gel-coated aluminum plates (60 F_254_, E. Merck). Column chromatography was performed on silica gel (200–300 mesh). Gel filtration was performed on Sephadex LH-20 (Pharmacia). Optical rotations were obtained on a Hanon P850 Automatic Polarimeter. Experiments under ultraviolet (UV) irradiation were carried out using a safe and stable mercury lamp spotlight system purchased from Beijing Zhongjiao Jinyuan Technology Co., Ltd. (Item No. CEL-M500). ^1^H NMR spectra were recorded at room temperature for solutions in CDCl_3_ or D_2_O with Avance III-400 or III-600 instruments (Bruker), and the chemical shifts were referenced to the peak for TMS (0 ppm, CDCl_3_) or external CH_3_OH (3.34 ppm, D_2_O). ^13^C NMR spectra were recorded using the same NMR spectrometers, and the chemical shifts were reported relative to the internal CDCl_3_ (δ = 77.16 ppm) or external CH_3_OH (49.70 ppm, D_2_O). High-resolution mass spectrometry (HRMS) experiments were performed on a Waters Xevo G2 Q-TOF spectrometer or a Bruker APEX IV FTMS instrument.

#### 3.1.1. Synthesis of Compound **1-1**

Compound **2** [27] (40.0 mg, 9.7 μmol) was dissolved in H_2_NCH_2_CH_2_NH_2_/*n*-BuOH (*v*/*v* = 1:3, 20 mL). The solution was heated at 120 °C for 6 h. The solution was concentrated under reduced pressure. The residue was dissolved in MeOH (5.0 mL) and then Ac_2_O (1.0 mL) was added, and the reaction was performed at room temperature for 3 h. The solution was concentrated under reduced pressure. The residue was dissolved in THF/MeOH (*v*/*v* = 1:4, 5 mL), and then LiOH (50.0 mg, 1.2 mmol) was added. The mixture was heated at 80 °C overnight. H^+^ resin was used for neutralization. The mixture was filtered, and the filtrate was concentrated under reduced pressure. The crude product was purified via column chromatography on silica gel (toluene: CH_3_CN = 6:1, *v*/*v*) to afford white solids. The obtained product was dissolved in CH_2_Cl_2_/MeOH/H_2_O (*v*/*v*, 3:3:1, 7 mL), and then Pd(OH)_2_ (10%, 15.0 mg) was added. The reaction was carried out under a hydrogen atmosphere of 40 psi for 30 h. The mixture was filtered, and the filtrate was concentrated under reduced pressure. The residue was purified using column chromatography on gel LH20 (H_2_O: MeOH = 15:1, *v*/*v*) to afford **1-1** as white solids (10.0 mg, 63% yield).

^1^H NMR (400 MHz, D_2_O) δ 5.19 (d, *J* = 3.1 Hz, 1H), 5.03 (t, *J* = 3.6 Hz, 2H), 4.83–4.76 (m, 1H), 4.62 (d, *J* = 8.0 Hz, 2H), 4.45–4.31 (m, 4H), 4.19–4.11 (m, 1H), 4.07 (d, *J* = 3.3 Hz, 1H), 4.00 (d, *J* = 3.3 Hz, 1H), 3.95–3.30 (m, 46H), 3.24–3.16 (m, 1H), 2.92 (d, *J* = 7.5 Hz, 2H), 1.93 (s, 6H), 1.65–1.54 (m, 4H), 1.43–1.30 (m, 2H), 1.17 (d, *J* = 6.6 Hz, 3H), 1.15 (d, *J* = 6.7 Hz, 3H), 1.06 (d, *J* = 6.5 Hz, 3H). ^13^C NMR (151 MHz, D_2_O) δ 174.70, 174.66, 102.95, 102.50, 102.42, 101.99, 101.72, 100.20, 99.43, 98.69, 98.58, 82.08, 81.55, 78.40, 76.38, 75.32, 75.11, 74.86, 74.77, 74.45, 73.56, 73.04, 72.80, 71.94, 71.85, 71.70, 70.56, 70.08, 69.94, 69.73, 69.17, 68.75, 68.28, 68.21, 67.70, 67.65, 66.92, 66.79, 66.69, 61.47, 61.41, 61.27, 61.11, 60.95, 60.08, 59.78, 59.65, 48.86, 39.36, 28.14, 26.43, 22.24, 22.07, 15.45, 15.43, 15.26. HRMS (ESI): [M + H]^+^ C_63_H_110_N_3_O_43_^+^ *m*/*z* calcd. 1596.65, found 1596.64. The data are consistent with those reported previously [27].

#### 3.1.2. Synthesis of Compound **1-2**

Compound **2** (40.0 mg, 9.7 μmol) was dissolved in H_2_NCH_2_CH_2_NH_2_/*n*-BuOH (*v*/*v* = 1:3, 20 mL). The solution was heated at 120 °C for 6 h. The solution was concentrated under reduced pressure. The residue was dissolved in THF/MeOH (*v*/*v* = 1:4, 5 mL), and then LiOH (50.0 mg, 1.2 mmol) was added. The solution was heated at 80 °C overnight. The solution was concentrated under reduced pressure. Then, the residue was dissolved in CH_2_Cl_2_ and washed with water, and then it was concentrated under reduced pressure and further dried under vacuum.

The residue was dissolved in MeOH (5.0 mL), and the reaction mixture was stirred for 10 min. 4-Dimethylaminopyridine (600 mg) was dissolved in MeOH (5.0 mL), stirred for 10 min, and then added to the reaction mixture. The reaction mixture was stirred for 20 min at room temperature and then cooled to 0 °C for 10 min. CF_3_COOCH_3_ (16 mL in total) was added 4 times every 5 min, and it was stirred at room temperature for 48 h. The mixture was concentrated under reduced pressure and further dried under vacuum.

The crude product was purified via column chromatography on silica gel (toluene: CH_3_CN = 6:1, *v*/*v*) to afford white solids. The obtained product was dissolved in CH_2_Cl_2_/MeOH/H_2_O (*v*/*v*, 3:3:1, 7 mL), and then Pd(OH)_2_ (10%, 15.0 mg) was added. The reaction was carried out under a hydrogen atmosphere of 40 psi for 30 h. The mixture was filtered, and the filtrate was concentrated under reduced pressure. The residue was purified using column chromatography on gel LH20 (H_2_O: MeOH = 15:1, *v*/*v*) to afford **1-2** as white solids (6.4 mg, 43% yield).

^1^H NMR (400 MHz, D_2_O) δ 5.16 (d, *J* = 3.1 Hz, 1H), 4.91 (t, *J* = 3.4 Hz, 2H), 4.43–4.28 (m, 4H), 4.16–4.13 (m, 1H), 4.05 (d, *J* = 2.8 Hz, 1H), 4.01–3.34 (m, 47H), 3.20–3.14 (m, 1H), 2.88 (t, *J* = 7.5 Hz, 2H), 1.56 (q, *J* = 7.8 Hz, 4H), 1.39–1.29 (m, 2H), 1.15 (d, *J* = 6.5 Hz, 3H), 1.12 (d, *J* = 6.6 Hz, 3H), 1.01 (d, *J* = 6.5 Hz, 3H). ^13^C NMR (151 MHz, D_2_O) δ 159.75, 159.63, 159.50, 159.38, 159.25, 159.12, 159.00, 158.88, 138.14, 118.63, 116.73, 114.84, 112.94, 106.73, 102.94, 101.97, 101.83, 101.80, 101.71, 100.17, 99.40, 99.25, 99.00, 98.85, 82.45, 81.73, 78.36, 76.28, 75.38, 75.14, 74.86, 74.84, 74.74, 74.51, 74.42, 73.53, 73.06, 72.83, 72.79, 72.62, 71.92, 71.84, 71.70, 71.56, 71.20, 70.38, 70.07, 69.78, 69.73, 69.57, 69.53, 69.24, 68.76, 68.26, 68.16, 68.13, 68.09, 67.51, 67.44, 66.94, 66.90, 66.82, 61.51, 61.44, 60.93, 60.52, 60.05, 59.67, 59.52, 56.62, 56.45, 54.99, 39.34, 39.27, 28.13, 26.39, 22.06, 20.03, 15.45, 15.40, 15.19. MALDI-TOF-MS: [M + Na]^+^ C_63_H_103_N_3_O_43_F_6_Na^+^ *m*/*z* calcd. 1726.58, found 1726.67; [M + K] ^+^ C_63_H_103_N_3_O_43_F_6_K^+^ *m*/*z* calcd. 1742.55, found 1742.64.

#### 3.1.3. Synthesis of Compound **1-3**

Compound **2** (40.0 mg, 9.7 μmol) was dissolved in H_2_NCH_2_CH_2_NH_2_/*n*-BuOH (*v*/*v* = 1:3, 20 mL). The solution was heated at 120 °C for 6 h. The solution was concentrated under reduced pressure. The residue was dissolved in THF/MeOH (*v*/*v* = 1:4, 5 mL), and then LiOH (50.0 mg, 1.2 mmol) was added. The mixture was heated at 80 °C overnight. The mixture was concentrated under reduced pressure. Then, the residue was dissolved in CH_2_Cl_2_ and washed with water, and then it was concentrated under reduced pressure and further dried under vacuum.

The residue was dissolved in MeOH (5.0 mL), and the reaction mixture was stirred for 10 min. 4-Dimethylaminopyridine (600 mg) was dissolved in MeOH (5.0 mL), stirred for 10 min, and then added to the reaction mixture. The reaction mixture was stirred for 20 min at room temperature and then cooled to 0 °C for 10 min. CF_2_COOCH_3_ (16 mL in total) was added 4 times every 5 min, and it was stirred at room temperature for 48 h. The mixture was concentrated under reduced pressure and further dried under vacuum.

The crude product was purified via column chromatography on silica gel (toluene: CH_3_CN = 6:1, *v*/*v*) to afford white solids. The obtained product was dissolved in CH_2_Cl_2_/MeOH/H_2_O (*v*/*v*, 3:3:1, 7 mL), and then Pd(OH)_2_ (10%, 15.0 mg) was added. The reaction was carried out under a hydrogen atmosphere of 40 psi for 30 h. The mixture was filtered, and the filtrate was concentrated under reduced pressure. The residue was purified using column chromatography on gel LH20 (H_2_O: MeOH = 15:1, *v*/*v*) to afford **1-3** as white solids (5.5 mg, 36% yield).

^1^H NMR (400 MHz, D_2_O) δ 6.05 (t, *J* = 53.6 Hz, 2H), 5.17 (d, *J* = 3.2 Hz, 1H), 4.95 (t, *J* = 3.5 Hz, 2H), 4.43–4.28 (m, 4H), 4.18–4.12 (m, 1H), 4.05 (d, *J* = 3.3 Hz, 1H), 4.00–3.35 (m, 47H), 3.19 (t, *J* = 8.6 Hz, 1H), 2.90 (t, *J* = 7.5 Hz, 2H), 1.65–1.51 (m, 4H), 1.44–1.29 (m, 2H), 1.16 (d, *J* = 6.6 Hz, 3H), 1.13 (d, *J* = 6.6 Hz, 3H), 1.02 (d, *J* = 6.6 Hz, 3H). ^13^C NMR (151 MHz, D_2_O) δ 166.38, 166.32, 166.21, 166.15, 166.04, 165.98, 158.31, 138.85, 110.76, 110.71, 109.12, 109.07, 107.50, 107.47, 107.42, 103.70, 102.78, 102.75, 102.68, 102.54, 100.94, 100.18, 99.61, 99.49, 83.07, 82.53, 79.14, 77.08, 76.14, 75.92, 75.64, 75.63, 75.52, 75.30, 75.24, 75.20, 74.31, 73.66, 73.56, 73.50, 72.70, 72.62, 72.47, 71.17, 70.84, 70.64, 70.50, 69.95, 69.53, 69.23, 69.04, 68.94, 68.35, 68.29, 67.70, 67.63, 67.53, 62.27, 62.20, 61.71, 60.83, 60.48, 60.33, 57.00, 56.84, 47.44, 43.02, 40.11, 40.06, 28.91, 27.16, 22.84, 20.80, 16.22, 16.18, 16.00, 12.54, 11.28, 8.98. MALDI-TOF-MS: [M + Na]^+^ C_63_H_105_N_3_O_43_F_4_Na^+^ *m*/*z* calcd. 1690.60, found 1691.19; [M + K]^+^ C_63_H_105_N_3_O_43_F_4_K^+^ *m*/*z* calcd. 1706.57, found 1707.16.

#### 3.1.4. Synthesis of Compound **3-1**

4 Å MS (400 mg) was added to a 10 mL quartz two-necked reaction flask, baked at high temperature, and cooled under vacuum. Under Ar protection, compound **4** (20.0 mg, 17.6 μmol), Umemoto’s reagent (16.0 mg, 44. 1 µmol), Cu(OTf)_2_ (18.0 mg, 44.1 µmol), and anhydrous CH_2_Cl_2_ (4 mL) were added. The reaction mixture was stirred for 15 min and then cooled to −72 °C. The reaction flask was exposed to UV irradiation at −72 °C for 20 min. After the disappearance of **4** detected by TLC, the removal of UV irradiation was followed by the addition of a solution of compound **5** (15.4 mg, 14.7 µmol) and TTBP (6.6 mg, 26.4 µmol) in CH_2_Cl_2_ (0.5 mL) via syringe. The reaction mixture was stirred and slowly warmed to room temperature for 1.5 h. The reaction was cooled to −72 °C. Umemoto’s reagent (16.0 mg, 44.1 µmol) and Cu(OTf)_2_ (18.0 mg, 44.1 μmol) were added. The reaction flask was exposed to UV irradiation at −72 °C for 20 min. After the disappearance of the starting material detected by TLC, the removal of UV irradiation was followed by the addition of a solution of compound **6** (16.0 mg, 13.2 μmol) and TTBP (6.6 mg, 26.4 μmol) in CH_2_Cl_2_ (0.5 mL) via syringe. The reaction mixture was stirred and slowly warmed to room temperature for 1.5 h, and then quenched by Et_3_N (0.1 mL). The mixture was filtered and the filtrate was concentrated under reduced pressure. The crude product was purified by column chromatography on silica gel (petroleum ether: ethyl acetate = 1:1, *v*/*v*) to afford **3-1** as a white solid (50.0 mg, 61% yield).

${\left[\alpha \right]}_{D}^{25}$ -10.3 (c 0.24, CHCl_3_); ^1^H NMR (600 MHz, CDCl_3_) δ 7.71 (d, *J* = 7.2 Hz, 1H), 7.62 (d, *J* = 6.9 Hz, 1H), 7.56-7.49 (m, 4H), 7.46 (t, *J* = 7.2 Hz, 1H), 7.35-7.08 (m, 71H), 7.03 (t, *J* = 7.5 Hz, 2H), 6.82 (s, 3H), 5.66 (dd, *J* = 9.8, 9.1 Hz, 1H), 5.59 (dd, *J* = 10.0, 9.3 Hz, 1H), 5.40 (d, *J* = 8.2 Hz, 1H), 5.36 (d, *J* = 8.4 Hz, 1H), 5.16-5.12 (m, 4H), 5.03 (d, *J* = 11.6 Hz, 1H), 4.97 (d, *J* = 11.4 Hz, 1H), 4.84 (d, *J* = 11.4 Hz, 2H), 4.78–4.74 (m, 1H), 4.65–4.60 (m, 3H), 4.53–4.35 (m, 16H), 4.28 (d, *J* = 11.8 Hz, 1H), 4.21–4.11 (m, 9H), 3.98 (d, *J* = 11.8 Hz, 1H), 3.93–3.86 (m, 5H), 3.82–3.70 (m, 5H), 3.54-3.46 (m, 5H), 3.43-3.19 (m, 16H), 3.11 (m, 1H), 2.88 (d, *J* = 9.6 Hz, 1H), 2.78–2.74 (m, 1H), 2.65–2.60 (m, 1H), 2.48–2.44 (m, 1H), 2.42–2.10 (m, 12H), 1.67–1.66 (m, 9H), 1.55–1.43 (m, 4H), 1.26–1.21 (m, 2H). ^13^C NMR (151 MHz, CDCl_3_) δ 206.57, 206.38, 206.18, 171.62, 171.53, 171.04, 167.41, 167.38, 167.33, 167.05, 164.05, 156.65, 156.09, 139.35, 138.99, 138.97, 138.67, 138.49, 138.37, 138.29, 138.26, 137.95, 137.91, 137.85, 137.66, 136.81, 136.73, 133.54, 133.34, 133.23, 132.63, 131.22, 131.16, 131.08, 130.90, 129.69, 129.35, 128.51, 128.46, 128.43, 128.39, 128.35, 128.20, 128.18, 128.16, 128.12, 128.11, 128.06, 128.02, 127.99, 127.92, 127.86, 127.83, 127.80, 127.77, 127.72, 127.65, 127.60, 127.41, 127.38, 127.32, 127.30, 127.09, 126.94, 126.64, 126.35, 123.30, 123.20, 123.02, 122.85, 103.41, 102.27, 100.41, 100.31, 99.46, 99.27, 82.84, 82.19, 81.53, 80.69, 80.32, 78.52, 76.53, 76.27, 75.88, 75.29, 75.07, 74.96, 74.88, 74.83, 74.59, 74.48, 74.42, 74.37, 73.95, 73.92, 73.62, 73.42, 73.38, 73.36, 73.17, 73.13, 72.97, 72.82, 72.80, 72.32, 71.98, 71.76, 71.48, 70.33, 70.24, 69.57, 69.47, 68.25, 68.18, 67.98, 67.62, 67.45, 67.08, 55.05, 54.81, 50.47, 50.15, 47.10, 46.14, 37.75, 37.63, 37.62, 29.89, 29.30, 29.24, 27.82, 27.77, 23.29, 27.46, 23.25. HRMS (ESI): [M + 2NH_4_]^2+^ C_185_H_199_N_5_O_42_^2+^ *m*/*z* calcd. 1581.18, found 1581.19. The data are consistent with those reported previously [27].

#### 3.1.5. Synthesis of Compound **3-2**

Compound **3-1** (50.0 mg, 16 μmol) was dissolved in a solution of THF/MeOH (*v*/*v* = 10: 1, 4.4 mL). H_2_NNH_2_·HOAc (22.0 mg, 240 μmol) was added. The mixture was stirred at room temperature overnight. The mixture was diluted with CH_2_Cl_2_, washed sequentially with water, and NaCl aq., and dried over anhydrous Na_2_SO_4_. The solution was filtered and the filtrate was concentrated under reduced pressure. The crude product was purified by column chromatography on silica gel (petroleum ether: ethyl acetate: CH_2_Cl_2_ = 1:1:0.25, *v*/*v*/*v*) to afford **3-2** as a white solid (41.5 mg, 90% yield). 

${\left[\alpha \right]}_{D}^{25}$ −0.78 (c 0.44, CHCl_3_); ^1^H NMR (600 MHz, CDCl_3_) δ 7.76 (d, *J* = 7.6 Hz, 1H), 7.66–7.65 (m, 3H), 7.57–7.54 (m, 2H), 7.46 (t, *J* = 7.5 Hz, 1H), 7.38–7.08 (m, 74H), 7.03–7.00 (m, 4H), 6.91 (d, *J* = 7.6 Hz, 1H), 6.85–6.83 (m, 2H), 5.43 (dd, *J* = 9.0, 8.3 Hz, 1H), 5.31 (d, *J* = 8.4 Hz, 1H), 5.28 (d, *J* = 8.5 Hz, 1H), 5.13 (d, *J* = 9.5 Hz, 2H), 5.01 (d, *J* = 11.6 Hz, 2H), 4.97 (d, *J* = 11.4 Hz, 1H), 4.84 (d, *J* = 10.4 Hz, 1H), 4.81 (d, *J* = 11.4 Hz, 1H), 4.76 (t, *J* = 11.7 Hz, 1H), 4.68 (d, *J* = 12.1 Hz, 1H), 4.64–4.50 (m, 7H), 4.47–4.36 (m, 8H), 4.29–4.08 (m, 14H), 4.03–4.01 (m, 2H), 3.93–3.86 (m, 5H), 3.82–3.73 (m, 6H), 3.65–3.63 (m, 1H), 3.60–3.36 (m, 11H), 3.32–3.17 (m, 10H), 3.12–3.10 (m, 1H), 2.91–2.89 (m, 1H), 2.62–2.61 (m, 1H), 1.55–1.42 (m, 4H), 1.30-1.21 (m, 2H). ^13^C NMR (151 MHz, CDCl_3_) δ 167.69, 167.65, 167.60, 167.28, 164.24, 156.64, 156.07, 139.38, 139.00, 138.67, 138.45, 138.39, 138.33, 138.28, 138.17, 138.06, 137.84, 137.79, 137.31, 137.23, 136.79, 136.70, 133.44, 133.33, 132.83, 131.42, 131.35, 131.12, 129.74, 129.34, 128.69, 128.53, 128.44, 128.36, 128.26, 128.20, 128.18, 128.15, 128.14, 128.11, 128.09, 128.04, 128.01, 127.92, 127.84, 127.80, 127.74, 127.72, 127.64, 127.62, 127.59, 127.52, 127.49, 127.45, 127.38, 127.29, 127.26, 127.07, 126.97, 126.90, 126.64, 126.38, 123.30, 122.98, 122.95, 122.59, 104.16, 103.39, 102.25, 101.64, 99.74, 99.42, 83.31, 82.83, 81.99, 81.95, 81.89, 81.50, 80.31, 78.60, 76.63, 75.90, 75.44, 75.27, 74.87, 74.80, 74.59, 74.55, 74.43, 73.97, 73.81, 73.46, 73.44, 73.41, 73.32, 73.09, 72.96, 72.83, 72.63, 72.44, 72.34, 71.13, 70.95, 69.53, 69.46, 69.18, 68.99, 68.89, 68.40, 68.36, 68.28, 67.58, 67.06, 56.40, 56.01, 50.46, 50.14, 47.09, 46.13, 29.31, 29.27, 29.22, 27.85, 27.44, 23.29, 23.24. HRMS (ESI): [M + 2NH_4_]^2+^ C_170_H_181_N_5_O_36_^2+^ *m*/*z* calcd. 1434.12, found 1434.13. The data are consistent with those reported previously [27].

#### 3.1.6. Synthesis of Compound **2**

4 Å MS (1.5 g) was baked at high temperature and cooled under vacuum, then was added to a 25 mL quartz three-necked reaction flask. Under Ar protection, donor **7** (30.0 mg, 54.1 μmol) was added. Umemoto’s reagent (64.0 mg, 0.16 mmol) and Cu(OTf)_2_ (59.0 mg, 0.16 mmol) were dissolved or suspended in anhydrous CH_2_Cl_2_ (7.0 mL) under ultrasound and then added via syringe. The reaction mixture was stirred for 5 min and then cooled to −50 °C. The reaction flask was exposed to UV irradiation for 4 min. The removal of UV irradiation was followed by the addition of a solution of the acceptor **3-2** (13.0 mg, 5.4 µmol) and TTBP (20.0 mg, 81.1 µmol) in CH_2_Cl_2_ (2.0 mL) via syringe. The reaction mixture was stirred and slowly warmed to room temperature in 2.5 h, and then quenched by Et_3_N (0.1 mL). The mixture was filtered and the filtrate was concentrated under reduced pressure. The residue was purified by column chromatography on silica gel (petroleum ether: ethyl acetate = 2:1, *v*/*v*) to afford **2** as a white solid (36.0 mg, 90% yield). 

${\left[\alpha \right]}_{D}^{25}$ −5.21 (c 0.37, CHCl_3_); ^1^H NMR (600 MHz, CDCl_3_) δ 8.05 (d, *J* = 7.2 Hz, 2H), 7.83 (d, *J* = 7.5 Hz, 2H), 7.72 (t, *J* = 7.8 Hz, 5H), 7.62–7.57 (m, 4H), 7.54–7.43 (m, 9H), 7.36–7.03 (m, 96H), 7.00–6.94 (m, 5H), 6.83–6.82 (m, 2H), 6.76–6.70 (m, 4H), 6.54 (d, *J* = 7.0 Hz, 2H), 6.45 (d, *J* = 7.5 Hz, 2H), 5.70 (d, *J* = 2.3 Hz, 1H), 5.67 (d, *J* = 3.8 Hz, 1H), 5.64–5.63 (m, 2H), 5.29 (dd, *J* = 8.7, 8.0 Hz, 1H), 5.19 (d, *J* = 8.5 Hz, 1H), 5.13 (d, *J* = 6.5 Hz, 2H), 5.07-5.00 (m, 4H), 4.95 (dd, *J* = 13.2, 6.5 Hz, 1H), 4.85–4.48 (m, 28H), 4.45–4.28 (m, 10H), 4.25–4.05 (m, 15H), 4.03–3.94 (m, 6H), 3.91–3.88 (m, 2H), 3.84–3.69 (m, 7H), 3.57–3.19 (m, 23H), 3.11 (d, *J* = 9.5 Hz, 2H), 2.86–2.84 (m, 1H), 1.54–1.44 (m, 4H), 1.27 (d, *J* = 6.5 Hz, 3H), 1.11 (d, *J* = 6.5 Hz, 3H), 0.98 (d, *J* = 6.5 Hz, 3H). ^13^C NMR (151 MHz, CDCl_3_) δ 168.33, 168.31,166.73, 166.69, 166.52, 165.91, 163.92, 156.66, 156.10, 139.36, 138.99, 138.69, 138.44, 138.41, 138.32, 138.23, 138.15, 138.09, 138.05, 137.99, 137.91, 137.88, 137.83, 137.61, 137.54, 137.33, 136.84, 136.75, 133.56, 133.07, 132.88, 132.44, 132.32, 131.68, 131.04, 130.53, 130.25, 130.20, 129.96, 129.94, 129.86, 129.74, 129.72, 129.48, 129.39, 128.55, 128.53, 128.47, 128.43, 128.38, 128.35, 128.33, 128.21, 128.20, 128.15, 128.12, 128.06, 128.02, 127.99, 127.94, 127.87, 127.86, 127.82, 127.78, 127.77, 127.74, 127.69, 127.67, 127.64, 127.60, 127.54, 127.50, 127.46, 127.43, 127.37, 127.32, 127.29, 127.27, 127.24, 127.18, 127.13, 127.00, 126.99, 126.92, 126.90, 126.86, 126.70, 126.22, 126.14, 123.26, 103.45, 102.59, 100.12, 99.93, 99.91, 99.59, 98.41, 98.20, 97.92, 83.90, 82.86, 81.81, 81.54, 80.05, 78.30, 77.42, 76.68, 76.06, 75.95, 75.81, 75.37, 74.98, 74.85, 74.62, 74.21, 74.11, 73.92, 73.72, 73.65, 73.52, 73.32, 73.23, 73.15, 73.12, 72.91, 72.87, 72.84, 72.76, 72.58, 72.50, 72.47, 71.98, 71.75, 71.71, 71.60, 71.56, 71.48, 71.23, 71.17, 71.08, 71.01, 69.63, 69.49, 68.21, 68.02, 67.74, 67.61, 67.59, 67.09, 65.27, 65.23, 65.22, 56.93, 56.76, 50.50, 50.19, 47.12, 46.16, 29.33, 27.88, 27.48, 23.31, 15.94, 15.85, 15.43. HRMS (ESI): [M + 2NH_4_]^2+^ C_251_H_259_N_5_O_51_^2+^ *m*/*z* calcd. 2079.39, found 2079.39. The data are consistent with those reported previously [27].

#### 3.1.7. Synthesis of CRM197 Glycoconjugates

Compound **8** (20 mg) was dissolved in DMF (150 μL) in a micro-centrifuge tube, and then triethylamine (8–10 μL) was added. The color of the solution turned yellow. Nonasaccharide (3 mg) was dissolved in DMF (150 μL) in a micro-centrifuge tube, and then it was slowly added to the solution of compound **8**. The micro-centrifuge tube was washed for dissolving the nonasaccharide with DMF (100 μL), and then it was also added to the reaction mixture and mixed well. The reaction mixture was stirred for 4 h at room temperature. The reaction solution was transferred to a micro-thick-walled reaction vial, and then it was concentrated under reduced pressure and further dried under vacuum. After that, the residue was dissolved in water, and it was washed with CH_2_Cl_2_. The aqueous phase was evaporated at room temperature. The residue was dissolved in 0.01 M PBS buffer (600 μL, pH 7.6), and a 0.01 M PBS solution of protein CRM197 (400 μL, 10 mg/mL) was added. Then, the solution was rotated at room temperature without mechanical stirring for 24 h. After that, the solution was centrifuged (4 °C, 12,000 *g*) with an ultrafiltration centrifuge tube (Millipore^®^, 0.5 mL, 30 kD) for 5 min and washed three times with ultrapure water. The remaining samples in the ultrafiltration centrifuge tube were diluted and taken out to obtain an aqueous solution containing CRM197 glycoconjugates.

#### 3.1.8. Synthesis of BSA Glycoconjugates

Compound **8** (10 mg) was dissolved in DMF (100 μL) in a micro-centrifuge tube, and then triethylamine (8–10 μL) was added. The color of the solution turned yellow. Nonasaccharide (1.5 mg) was dissolved in DMF (60 μL) in a micro-centrifuge tube, and then it was slowly added to the solution of compound **8**. The micro-centrifuge tube was washed for dissolving the nonasaccharide with DMF (60 μL), and then it was also added to the reaction mixture and mixed well. The reaction mixture was stirred for 4 h at room temperature. The reaction solution was transferred to a micro-thick-walled reaction vial, and then it was concentrated under reduced pressure and further dried under vacuum. After that, the residue was dissolved in water, and it was washed with CH_2_Cl_2_. The aqueous phase was evaporated at room temperature. The residue was dissolved in 0.01 M PBS buffer (300 μL, pH 7.6), and a 0.01 M PBS solution of protein BSA (200 μL, 10 mg/mL) was added. Then, the solution was rotated at room temperature without mechanical stirring for 24 h. After that, the solution was centrifuged (4 °C, 12,000 *g*) with an ultrafiltration centrifuge tube (Millipore^®^, 0.5 mL, 30 kD) for 5 min, and it was washed three times with ultrapure water. The remaining samples in the ultrafiltration centrifuge tube were diluted and taken out to obtain an aqueous solution containing BSA glycoconjugates.

### 3.2. Immunization of Mice

Groups of six mice (female pathogen-free BALB/c, age 6–8 weeks, Number, SCXKjing2016-0010, from the Department of Laboratory Animal Science, Peking University Health Science Center) were immunized four times at a two-week interval with unmodified KH-1-CRM197 or modified glycoconjugates (KH-1-DF-CRM197 and KH-1-TF-CRM197) (each containing 2 μg of carbohydrate in PBS, which was the diluent of the mother liquor mentioned in Table 1). Each vaccination was conducted through a subcutaneous (s.c.) injection with a mixture of the adjuvant (first with Freund’s complete adjuvant and then Freund’s incomplete adjuvant, Sigma). The mice were bled via the tail vein 1 day before the first vaccination, 13 days after the third vaccination, and 14 days after the fourth vaccination. Blood was clotted to obtain sera, which were stored at −80 °C. On day 56, the mice were euthanized for a series of analyses described below. The animals used in this study were well cared for, and the experiments were approved by the Peking University Health Science Center (LA2017096).

### 3.3. Serological Assays

The antigen-specific antibody titers of the sera were assessed using ELISA. An ELISA plate was coated with 100 μL of KH-1-BSA (including 0.02 μg of carbohydrate) overnight at 4 °C (0.1 M bicarbonate buffer, pH = 9.6). After being washed three times with PBST (0.05% Tween-20 in PBS), micro-wells were blocked with 3% BSA (200 μL/well). The original serum was serially diluted with 1% BSA. After the plate was washed, the serially diluted sera were added to the micro-wells (100 μL/well) and incubated for 1 h at 37 °C. For the IgG subclass assay, diluted sera (1:1000, from 13 days after the third vaccination) were added instead. Then, the plate was washed and incubated with a 1:5000 dilution of horseradish-peroxidase-conjugated goat anti-mouse IgG, IgG1, IgG2a, IgG2b, IgG3, or IgM (Southern Biotechnology Associates, Inc., Birmingham, AL, USA) for 1 h at 37 °C. Finally, the *o*-phenylenediamine (OPD) substrate was added to the plate in the dark for 15 min; the reaction was terminated by 1 M H_2_SO_4_ and then read at 490 nm. The antibody titer was defined as the highest dilution showing an absorbance of 0.1, after subtracting background. Meanwhile, the anti-modified-KH-1 antibody titers (sera from 14 days after the fourth vaccination) were determined using ELISA, with the plate coated by the corresponding modified KH-1-BSA conjugates instead.

### 3.4. Flow Cytometry

The binding of antisera to MCF-7 human breast cancer cells that highly expressed native KH-1 was tested through flow cytometry. Firstly, MCF-7 cells (5 × 10^5^ cells/well) were washed in PBS with a 3% fetal bovine serum and incubated with a test serum (25 μL, finally diluted 1/20, from preimmunization and 14 days after the fourth vaccination) for 30 min on ice. Then, a goat anti-mouse IgG antibody labeled with AF405 (100 μL, diluted 1/500) was added and incubated at room temperature for 30 min. The MFI of the stained cells was analyzed using a FACScan (Becton Dickinson).

### 3.5. Complement-Dependent Cytotoxicity (CDC) Assay

CDC was assayed on MCF-7 cells that highly expressed the KH-1 antigen with a non-radioactive cytotoxicity assay kit (Promega). MCF-7 cells (25 μL, 1 × 10^4^ cells/well) were seeded into 96-well plates (Corning). Diluted sera (from preimmunization and 14 days after the fourth vaccination) were added and incubated at 37 °C for 2 h (the final concentration of sera was 1:20). After washing the cells twice, a rabbit complement (1:20) was added to the cells and incubated at 37 °C for another 4 h. After centrifugation, the cell supernatants (50 μL/well) were isolated and transferred to another 96-well plate. LDH assay reagents (50 μL/well) were added and incubated at room temperature protected from light for 30 min, and then a stop solution (1 M H_2_SO_4_, 50 µL/well) was added to each well of the plate. The absorptions of these plates were read at 490 nm wavelength using a microplate reader. The assays were performed in triplicate. The percentage of cell lysis was calculated according to the following formula:

(1) $$Cell\;lysis\left(\%\right)=\frac{Experimental-Target\;Spontaneous}{Target\;Maxium-Target\;Spontaneous\;}\times 100\%$$

## 4. Conclusions

In conclusion, a native KH-1 antigen and two fluorine-modified KH-1 antigens were designed and synthesized, and the antigens were conjugated with the CRM197 protein for vaccination. The results showed that fluoro-modifications on the KH-1 antigen, especially KH-1-TF, significantly enhanced the immunogenicity of the KH-1 antigen. KH-1-TF-CRM197 elicited higher titers of anti-KH-1 IgG and IgM antibodies than the unmodified KH-1-CRM197. The IgG antibody elicited by KH-1-TF-CRM197 showed a stronger CDC effect than that of KH-1-CRM197 to KH-1-positive tumor cells. Moreover, KH-1-TF-CRM197 elicited a mixed Th1/Th2 response, which is helpful for eliminating tumor cells. These results are encouraging because they lay a foundation for improving the efficacy of KH-1 conjugate and prove the feasibility of the fluorine modification strategy for the development of carbohydrate-based antitumor vaccines, thus holding potential for effective cancer immunotherapy.

## Figures and Tables

**Table 1:** Carbohydrate loading levels of glycoconjugates.

Conjugate	Molecular Weight of Glycoconjugate	Total Molecular Weight of Carbohydrate on Conjugate	Molecular Weight of Single Nonasaccharide	Concentration of Carbohydrate (μg/mL)	Concentration of Protein (μg/mL)	Number of Nonasaccharide	Percentage of Carbohydrate Loading
KH-1-CRM197	67,285	8968	1596	213	1600	5.62	13.32%
KH-1-TF-CRM197	67,908	9591	1704	165	1167	5.63	14.12%
KH-1-DF-CRM197	70,134	11,817	1668	195	1145	7.08	16.85%
KH-1-BSA	80,787	14,350	1596	206	1156	8.99	17.76%
KH-1-TF-BSA	83,936	17,499	1704	221	1057	10.27	20.85%
KH-1-DF-BSA	79,742	13,305	1668	197	1079	7.98	16.69%

**Table 2:** Titers of the antibodies elicited by vaccines.

ELISA Titer Anti-KH-1 and Ratio of IgG/IgM after the Third and the Fourth Vaccination							ELISA Titer Anti-Modified-KH-1
Vaccine	After the Third Vaccination			After the Fourth Vaccination			After the Fourth Vaccination
	IgG	IgM	IgG/IgM	IgG	IgM	IgG/IgM	IgG*
KH-1-CRM197/FA	<100	242	-	45,268	8952	5.06	45,268
KH-1-DF-CRM197/FA	11,686	4480	2.61	101,447	20,437	4.96	216,853
KH-1-TF-CRM197/FA	848,249	6307	134.49	1,180,228	9670	122.05	2,259,394

IgG is the IgG titer of the mixed serum from 6 mice of the same group against KH-1-BSA, whereas IgG* is the titer against the corresponding modified KH-1-BSA. Mouse serum IgG obtained from the preimmune condition could not be detected when diluted 1:100.

## Data Availability

The data presented in this study are available in this article and Supplementary Material, or on request from the corresponding author.

## Supplementary Materials

The following supporting information can be downloaded at: https://www.mdpi.com/article/10.3390/molecules28041934/s1. Table S1: Screening of reaction conditions for trifluoroacetylation; Figure S1. The MALDI-TOF-MS result of CRM197; Figure S2. The MALDI-TOF-MS result of KH-1-CRM197; Figure S3. The MALDI-TOF-MS result of KH-1-DF-CRM197; Figure S4. The MALDI-TOF-MS result of KH-1-TF-CRM197; Figure S5. The MALDI-TOF-MS result of BSA; Figure S6. The MALDI-TOF-MS result of KH-1-BSA; Figure S7. The MALDI-TOF-MS result of KH-1-DF-BSA; Figure S8. The MALDI-TOF-MS result of KH-1-TF-BSA; Figure S9. 1H NMR of compound 1-2(400 MHz, D2O); Figure S10. 13C NMR of compound 1-2(151 MHz, D2O); Figure S11. 1H NMR of compound 1-3(400 MHz, D2O); Figure S12. 13C NMR of compound 1-3(151 MHz, D2O).
