# Supplementary material for: Cancer Vaccines Based on Fluorine-Modified KH-1 Elicit Robust Immune Response
Source: Molecules. 2023 Feb 17;28(4):1934. doi: 10.3390/molecules28041934 (PMC9963332; doi:10.3390/molecules28041934)
Supplement: Supplementary file 1 [file molecules-28-01934-s001.zip › molecules-2186764-supplementary.pdf]

Supplementary Material

Cancer Vaccines Based on Fluorine-modified KH-1 Elicit Robust Immune Response

Yang Liu, Bo-Han Li, Xiu-Jing Zheng, De-Cai Xiong* and Xin-Shan Ye*

Correspondence: decai@bjmu.edu.cn, xinshan@bjmu.edu.cn

Table S1. Screening of reaction conditions for trifluoroacetylation.

Reactant	Additives/Solvents	Results
$F_3CCOOCOCF_3$	DCM	The raw material remain, and the main product is multi-position substitution product
	DMAP/Py	The product system is complex and cannot be analyzed
	Py	The product system is complex and cannot be analyzed
	MeOH	No reaction
	$(CH_3)_2CHOH$	No reaction
	$(CH_3)_3COH$	No reaction
	DMAP/MeOH	No reaction
	TEA/MeOH	No reaction
$F_3CCOOCH_3$	MeOH	No reaction
	TEA/MeOH	No reaction
	DMAP/MeOH	The main product is the target product
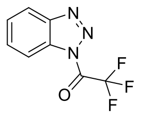	MeOH	No reaction
	DCM	A large amount of raw material remain, and the main product generated is not the target product
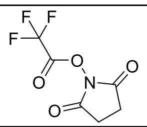	DCM	New product is formed, but it deteriorates quickly

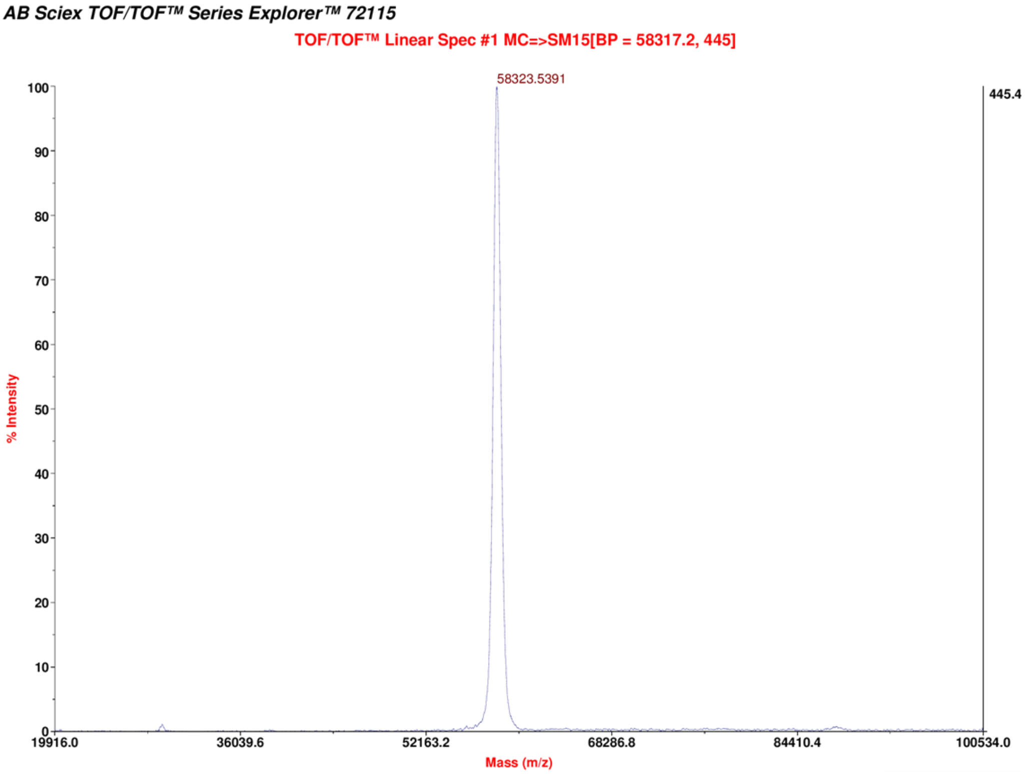

Figure S1. The MALDI-TOF-MS result of CRM197

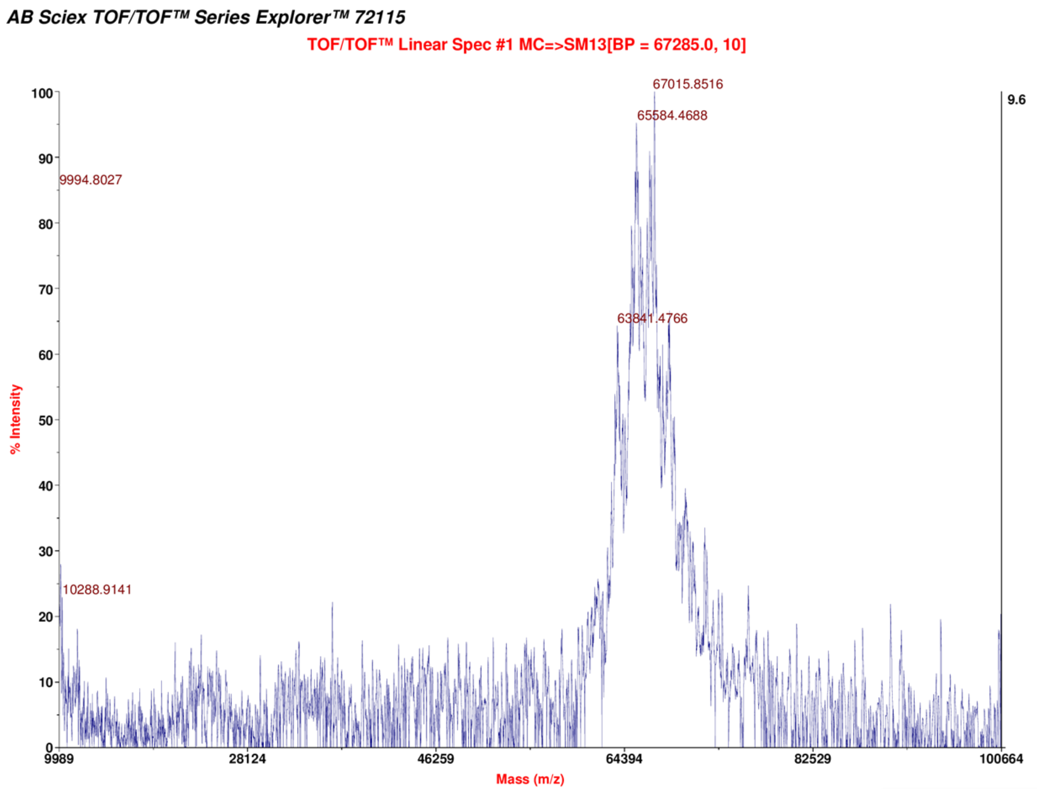

Figure S2. The MALDI-TOF-MS result of KH-1-CRM197

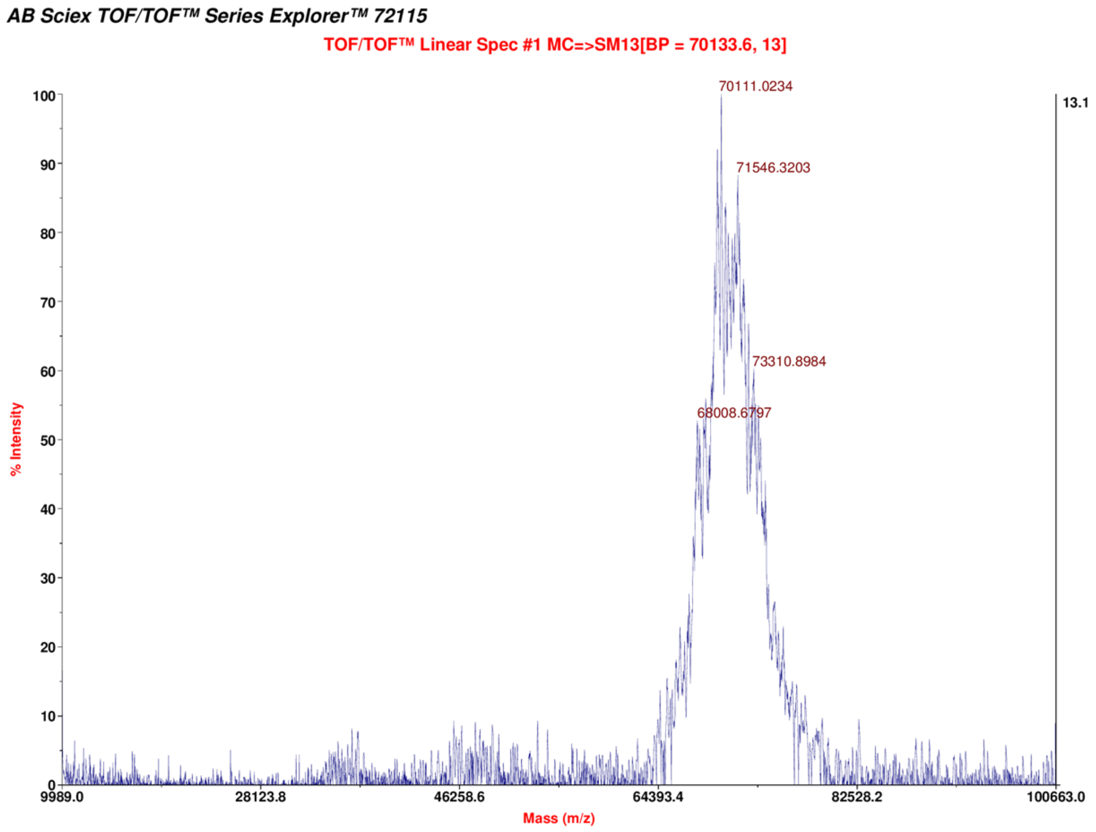

Figure S3. The MALDI-TOF-MS result of KH-1-DF-CRM197

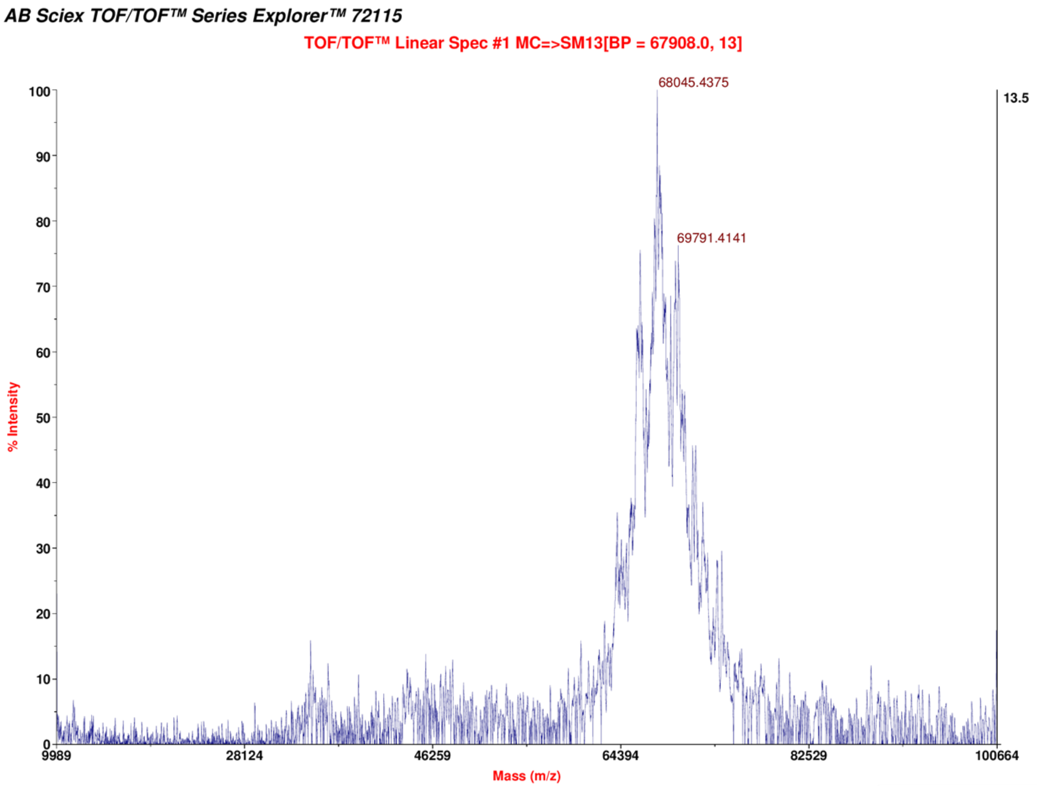

Figure S4. The MALDI-TOF-MS result of KH-1-TF-CRM197

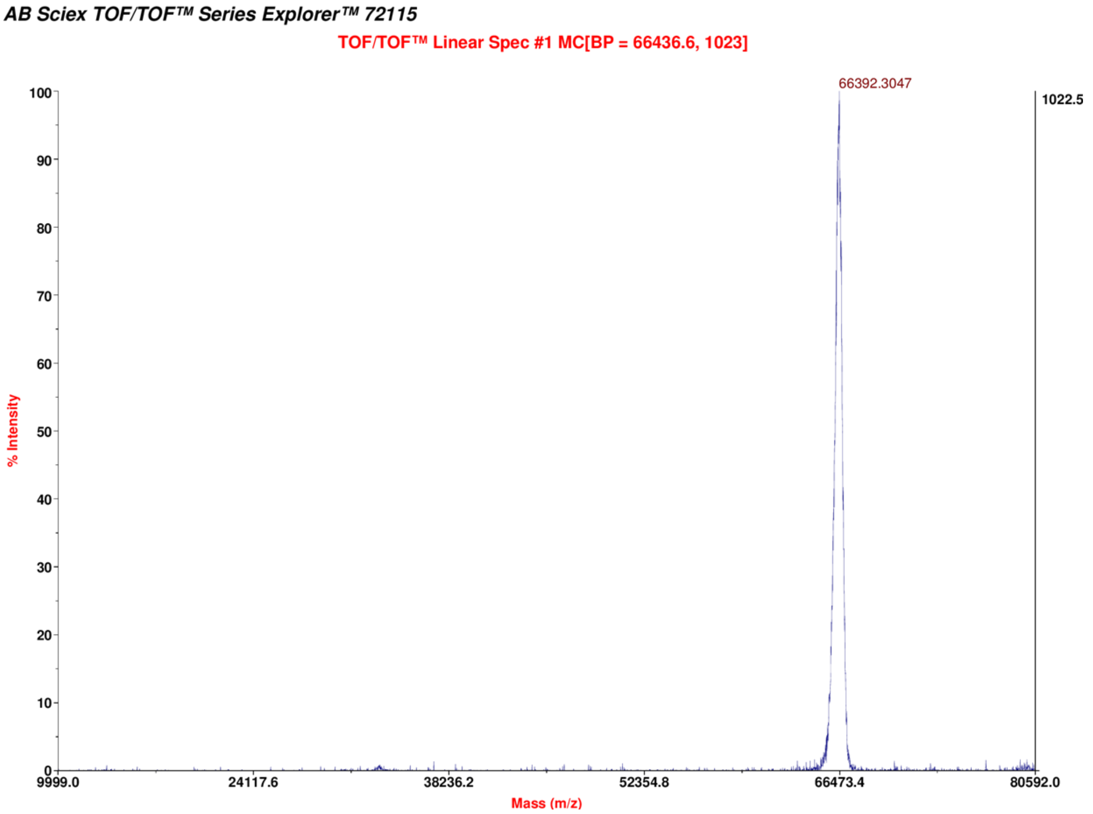

Figure S5. The MALDI-TOF-MS result of BSA

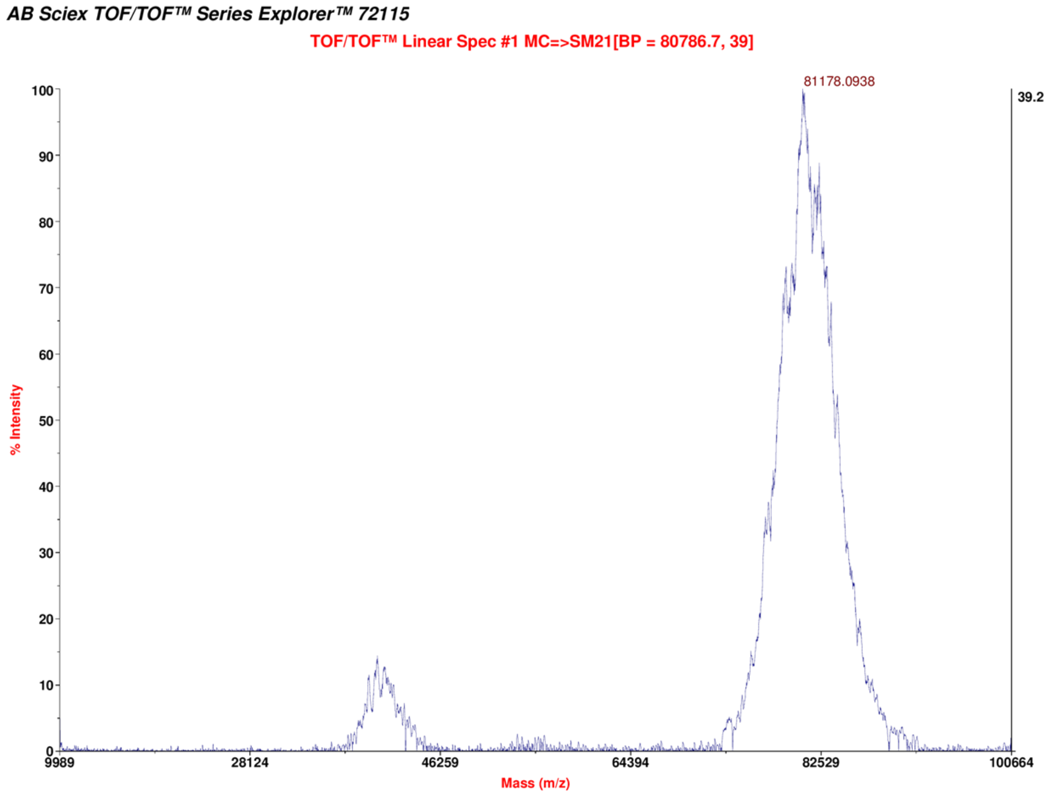

Figure S6. The MALDI-TOF-MS result of KH-1-BSA

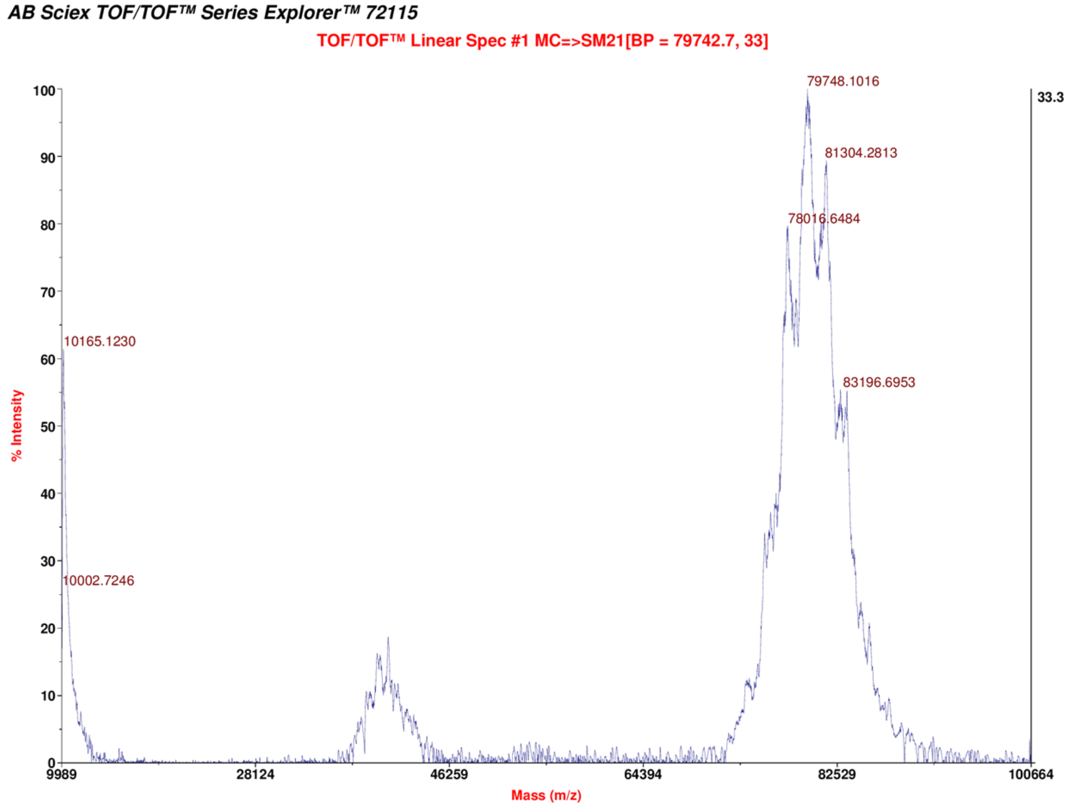

Figure S7. The MALDI-TOF-MS result of KH-1-DF-BSA

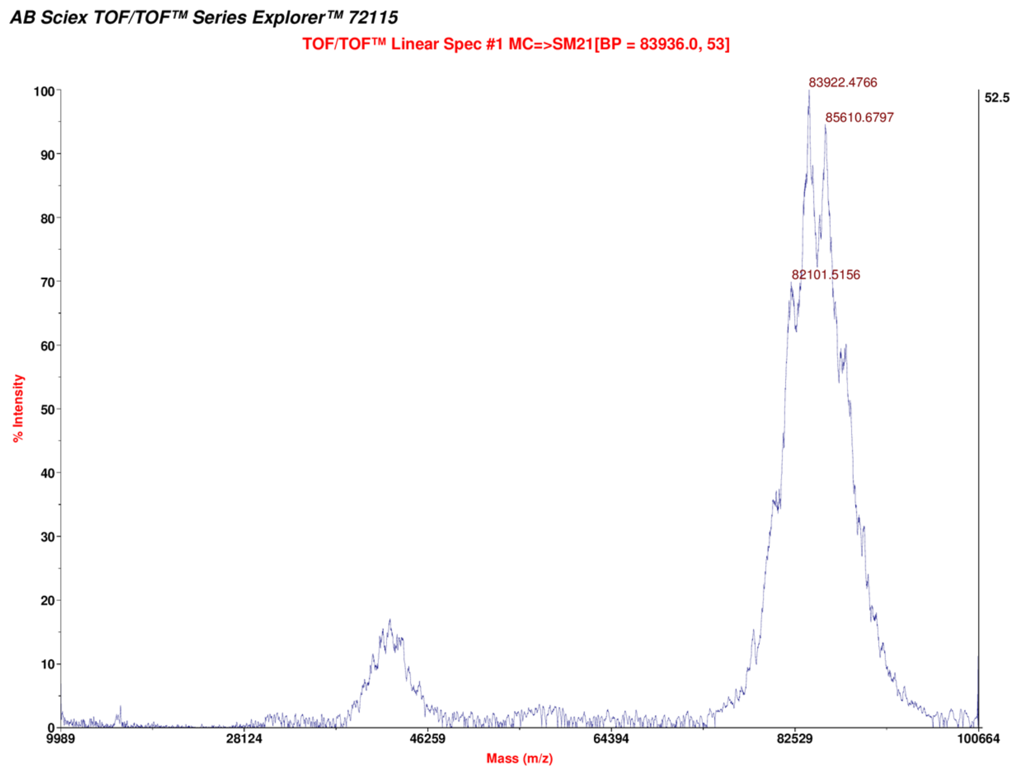

Figure S8. The MALDI-TOF-MS result of KH-1-TF-BSA

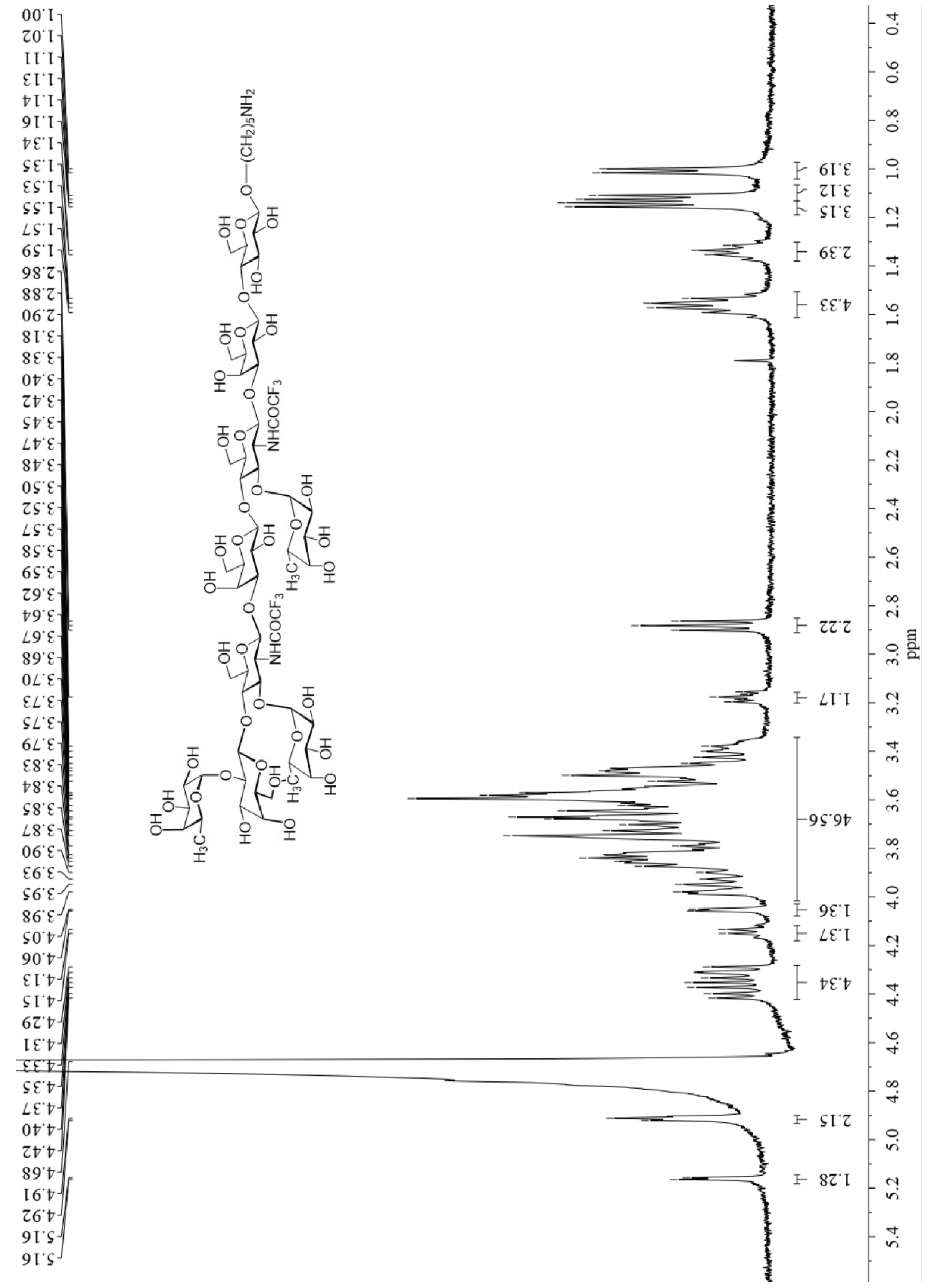

Figure S9. ^{1}H NMR of compound **1-2**（400 MHz, D_2O）

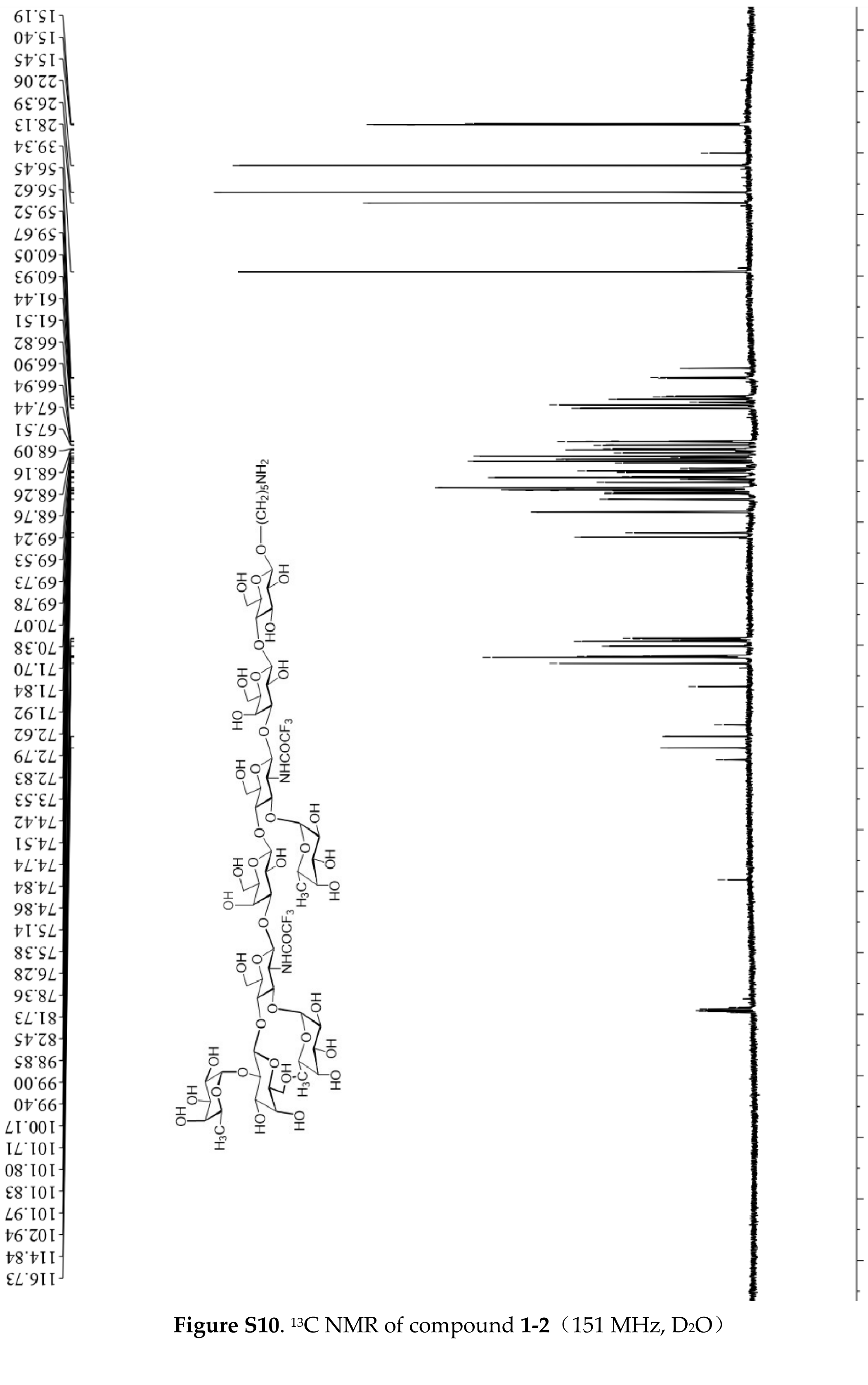

Figure S10. ^{13}C NMR of compound **1-2**（151 MHz, D_2O）

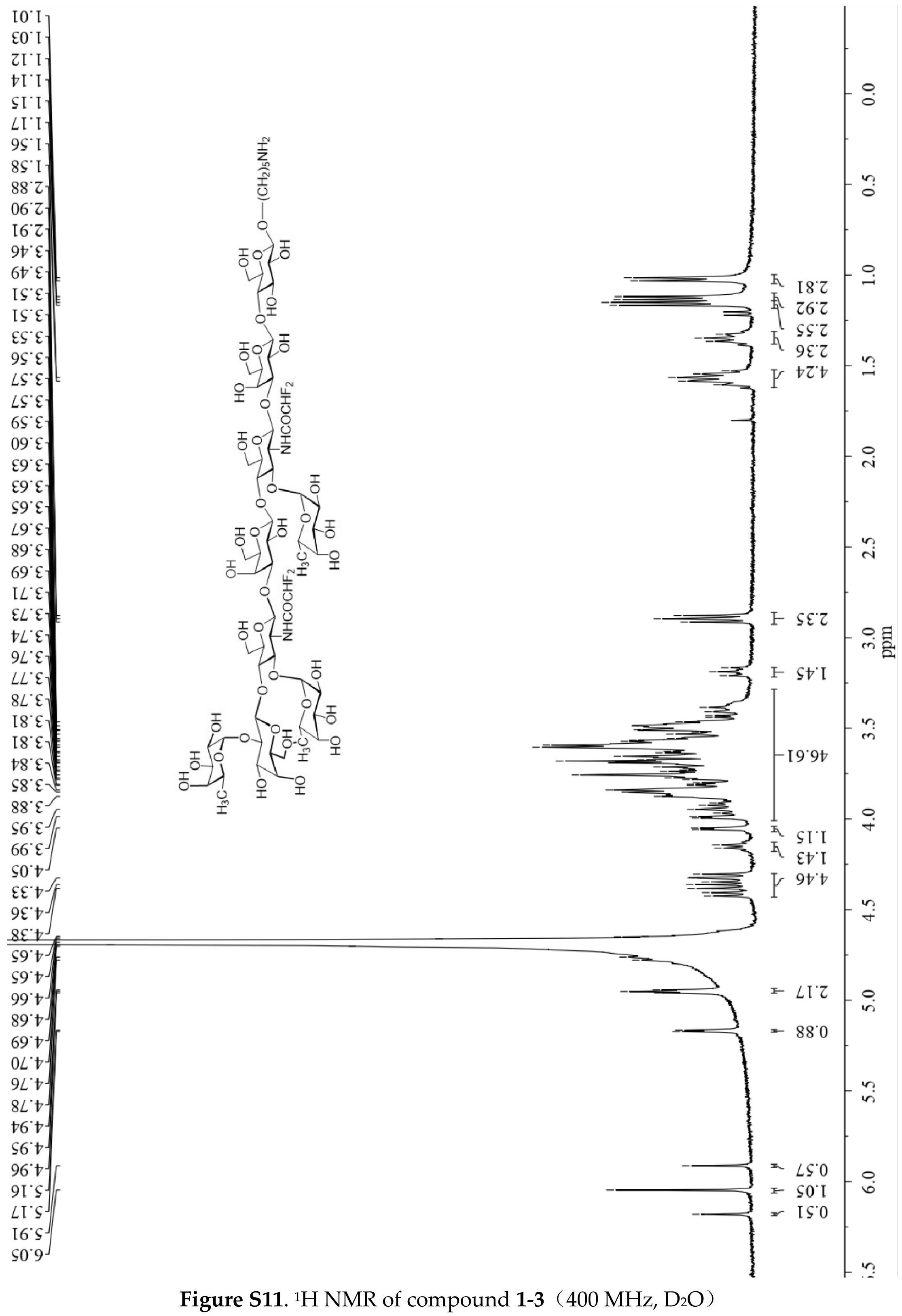

Figure S11. ^{1}H NMR of compound **1-3**（400 MHz, D_2O）

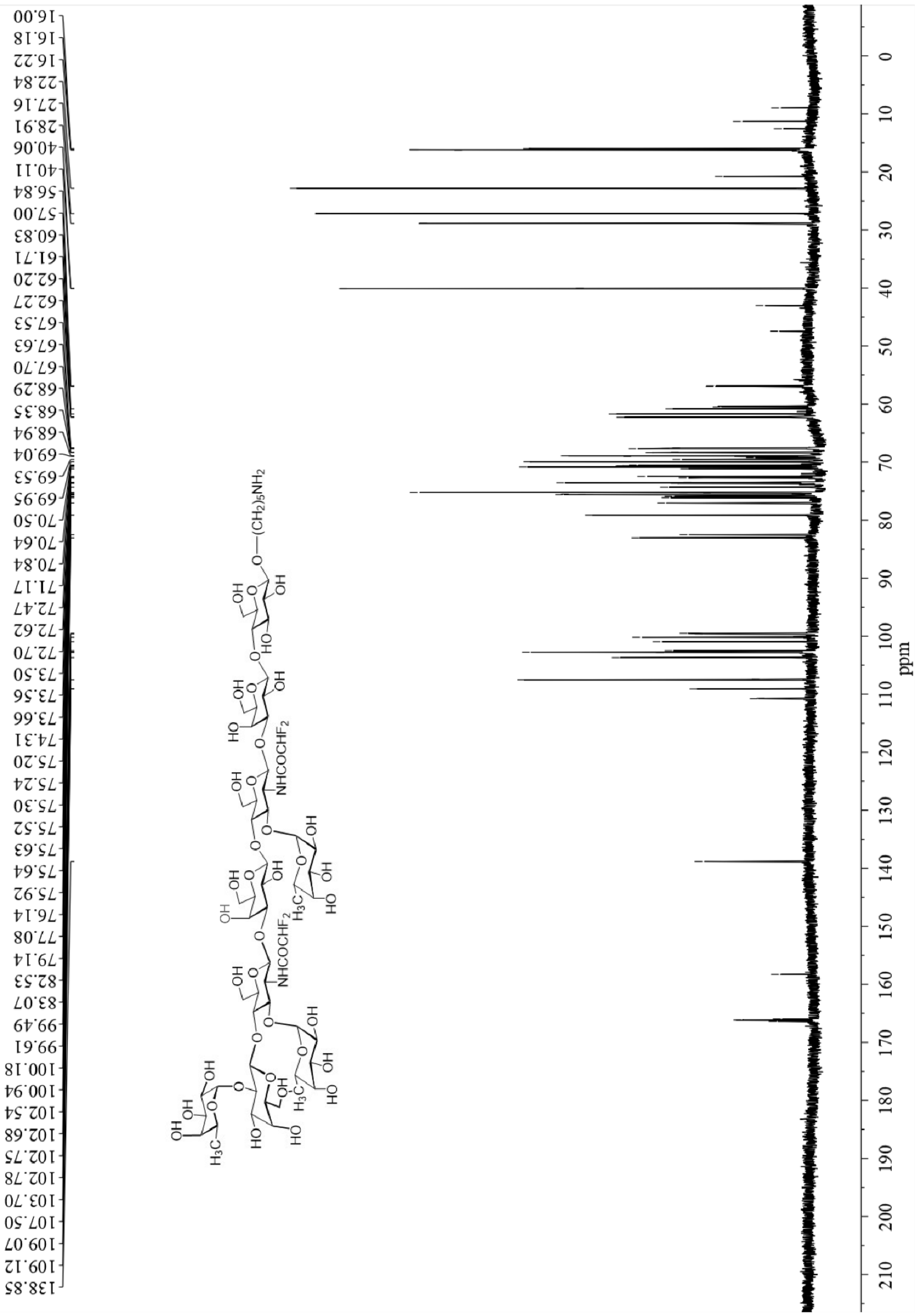

Figure S12. ^{13}C NMR of compound **1-3**（151 MHz, D_2O）
